# 60–700 K CTAT and PTAT Temperature Sensors with 4H-SiC Schottky Diodes

**DOI:** 10.3390/s21030942

**Published:** 2021-01-31

**Authors:** Razvan Pascu, Gheorghe Pristavu, Gheorghe Brezeanu, Florin Draghici, Philippe Godignon, Cosmin Romanitan, Matei Serbanescu, Adrian Tulbure

**Affiliations:** 1 National Institute for Research and Development in Microtechnologies—IMT Bucharest, 126A, Erou Iancu Nicolae Street, 077190 Bucharest, Romania; cosmin.romanitan@imt.ro; 2 Faculty of Electronics, Telecommunications and Information Technology, University “Politehnica” of Bucharest, 060042 Bucharest, Romania; gheorghe.pristavu@upb.ro (G.P.); gheorghe.brezeanu@dce.pub.ro (G.B.); florin.draghici@upb.ro (F.D.); matei.serbanescu@stud.etti.upb.ro (M.S.); 3 Romanian Young Academy, Research Institute of the University of Bucharest, University of Bucharest, 030018 Bucharest, Romania; 4 Centre Nacional de Microelectrònica, CNM-CSIC, 08193 Barcelona, Spain; philippe.godignon@cnm.es; 5 Department of Informatics, Mathematics and Electronics, Faculty of Exact Sciences and Engineering, University “1 Decembrie 1918” of Alba Iulia, No. 5, Gabriel Bethlen Street, 510009 Alba Iulia, Romania; aditulbure@uab.ro

**Keywords:** wide-range temperature sensor, SiC-Schottky diode, sensitivity, linearity, readout circuit

## Abstract

A SiC Schottky dual-diode temperature-sensing element, suitable for both complementary variation of V_F_ with absolute temperature (CTAT) and differential proportional to absolute temperature (PTAT) sensors, is demonstrated over 60–700 K, currently the widest range reported. The structure’s layout places the two identical diodes in close, symmetrical proximity. A stable and high-barrier Schottky contact based on Ni, annealed at 750 °C, is used. XRD analysis evinced the even distribution of Ni_2_Si over the entire Schottky contact area. Forward measurements in the 60–700 K range indicate nearly identical characteristics for the dual-diodes, with only minor inhomogeneity. Our parallel diode (*p-diode*) model is used to parameterize experimental curves and evaluate sensing performances over this far-reaching domain. High sensitivity, upwards of 2.32 mV/K, is obtained, with satisfactory linearity (R^2^ reaching 99.80%) for the CTAT sensor, even down to 60 K. The PTAT differential version boasts increased linearity, up to 99.95%. The lower sensitivity is, in this case, compensated by using a high-performing, low-cost readout circuit, leading to a peak 14.91 mV/K, without influencing linearity.

## 1. Introduction

Space missions, automotive, and various industries involve applications with a wide thermal variation and a large temperature range for detection. Here, temperature sensing plays a major role in ensuring safe operation or quality control capabilities. However, when working in such hostile environments, the performances of conventional sensing solutions can be affected by accuracy degradation or worse, general failure [1,2]. Usually, these detection systems include a series of commercial temperature sensors, which are based on thermocouples [3] or resistive temperature detectors [4,5]. Their accuracy and reliability are comparable, but neither are competitive with semiconductor-based temperature sensors [6,7], especially those fabricated on robust materials [8,9,10]. The increased request for high temperature-capable applications makes research in this domain constantly strive to find alternative solutions, which can satisfy specifications. However, electronic devices and systems based on conventional semiconductors, such as Si, are limited to operate at temperatures below 400 K [11]. On the other hand, wide-bandgap semiconductors have attracted much attention due to their electrical properties, together with their superior mechanical and chemical resilience. In particular, silicon carbide (SiC) has emerged as a viable alternative to replace Si in power and harsh-environment applications. SiC technology is very similar with that of Si and, in the last decade, its manufacturing processes have matured considerably, especially regarding the improvement in fabricated material defect density [12,13,14,15,16,17,18] and reliability of SiC-based devices [19,20,21,22,23,24,25,26]. In this regard, the simplest and most technologically mature device is the Schottky diode (SBD). When working as a temperature sensor [27], the key performances are linearity of the voltage-temperature dependence and long-term stability. The Schottky metal is also crucial, as the resulting contact’s barrier height (SBH) needs to be sufficient in order to ensure exponential current-voltage dependence for the forward characteristics over several orders of magnitude, at all temperatures of interest. In this sense, many literature contributions report metals such as Ti/Al [28], Ni [29], Pt [30] being used to achieve stable Schottky contacts on SiC. The most promising candidate for high temperature SiC Schottky diode-based sensors is Ni, due to its high work function and the capability to form very stable nickel silicide compounds on SiC after rapid post-metallization annealing in inert atmospheres [31]. It ensures a reasonably constant SBH, with values upwards of 1.73 V, for wide temperature ranges [25]. However, because of the detrimental effect of Schottky contact inhomogeneity [25,32,33,34,35,36,37,38], these indicators of merit degrade significantly for large temperature variations. As such, there are no reports of SiC-Schottky diodes working predictably over vast temperature ranges, much less so sensors. However, with the proposal of differential measurement techniques for SiC-Schottky diode temperature sensors [28,39], which considerably increase sensing linearity, as well as the recent introduction of a practical inhomogeneity modeling technique [40], the premises are set for investigating the potential performances of these devices over ranges spanning from cryogenic levels to high-temperature domains.

In this paper, we present wide-temperature sensing performances of a dual Schottky diode structure capable of working in either single or differential configurations. The structure is designed to operate at temperatures in the 60–700 K range. In order to increase sensitivity while maintaining linearity levels at an optimum, a simple and cost-effective readout circuit architecture is proposed and simulated for the differential topology.

## 2. Materials and Methods

### 2.1. Temperature Detection Methods Based on SiC Schottky Diodes

The forward voltage of Schottky diodes, biased at constant current, is given by the thermionic emission equation (neglecting the impact of the series resistance) [8,25]:

(1) $$V_{F}\approx n\Phi_{\mathrm{Bn},T}+nV_{\mathrm{th}}\ln\left(\frac{I_{F}}{A_{n}A_{S}T^{2}}\right)$$

where A_n_ is Richardson’s constant, A_S_ is the contact area, V_th_ is the thermal voltage, *n* is the ideality factor, and Φ_Bn,T_ is the conventional barrier. From Equation (1), a quasi-linear complementary variation of V_F_ with absolute temperature (CTAT) can be expressed, in respect to a reference (T_0_), thus:

(2) $$V_{F}\left(T\right)=n\Phi_{\mathrm{Bn},T}-\left[n\Phi_{\mathrm{Bn},T}+2nV_{\mathrm{th}0}\ln\left(\frac{T}{T_{0}}\right)-V_{F}\left(T_{0}\right)\right]\frac{T}{T_{0}}$$

V_th0_ is the thermal voltage associated with T_0_. From Equation (2), using Schottky diodes as CTAT sensors over moderate domains yields high sensitivities (in excess of 2 mV/K, depending on bias current levels, which determine V_F_(T_0_)) and reasonable linearity [24,28], while using simple and cost-effective readout circuits [8]. However, extending the operating temperature range evinces two significant causes for linearity degradation and sensitivity inconsistency:

- The innate variation of V_F_. Equation (2) contains a non-linearly temperature-dependent logarithmic term, which becomes significant when extending the T domain.
- Contact inhomogeneity. Analyzing Equation (2), it can be seen that Schottky diodes used for temperature sensing need to have constant barrier height and ideality factor values over the entire range of interest. Fluctuations in these parameters, primarily due to Schottky contact inhomogeneity, have been, however, ubiquitously reported [36,41,42,43,44,45]. The domain of variation for *n* and Φ_Bn,T_ is proportional with temperature range.

These sources of performance degradation can be mitigated using sensing methods based on differential forward voltage (∆V_F_) [39,46]. In contrast to the standard technique, ∆V_F_ can either increase (PTAT) or decrease (CTAT) with absolute temperature. Three ways of obtaining ∆V_F_ are discussed:

The single diode, dual current levels (SDDC) approach utilizes the voltage differential from a single diode, biased sequentially at two current levels, I_Fh_ > I_Fl_ (for the PTAT case):

(3) $$\Delta V_{F}\left(T\right)=V_{\mathrm{Fh}}-V_{\mathrm{Fl}}\overset{\left(1\right)}{\Rightarrow }\Delta V_{F}\left(T\right)=n\cdot V_{\mathrm{th}}\cdot \ln\left(\frac{I_{\mathrm{Fh}}}{I_{\mathrm{Fl}}}\right)=n\cdot \frac{k}{q}\cdot T\cdot \ln\left(\frac{I_{\mathrm{Fh}}}{I_{\mathrm{Fl}}}\right)$$

This expression for ∆V_F_(T) is directly proportional with temperature. The impact of contact inhomogeneity can also be alleviated by carefully tuning the I_Fh_ and I_Fl_ levels. Thus, SDDC is the technique that ensures best linearity. Sensitivity magnitude is proportional to the I_Fh_/I_Fl_ ratio. The downside is that the sequential biasing involves a readout circuit with a significantly more complex control loop. Acquiring the differential voltage requires either digital memory blocks or sample-and-hold cells, thereby delaying signal processing and increasing response times.

For the dual diode, single current level (DDSC) approach, the voltage difference is obtained from two diodes with different active areas (A_Sh_ > A_Sl_), biased at the same current:

(4) $$\Delta V_{F}=V_{\mathrm{Fh}}-V_{\mathrm{Fl}}\overset{\left(1\right)}{\Rightarrow }\Delta V_{F}\left(T\right)=n\cdot V_{\mathrm{th}}\cdot \ln\left(\frac{A_{\mathrm{Seffh}}}{A_{\mathrm{Seffl}}}\right)$$

This technique needs both diodes to have identical, temperature-invariable *n* and Φ_Bn,T_. In Equation (4), it is mandatory to use the effective contact areas (A_Seffh,l_), which generally differ greatly from their nominal values (A_Sh,l_). We presented procedures to evaluate A_Seff_ for Schottky diodes with non-uniform contacts (like Ni/SiC) [25,40]. While this technique requires readout circuits which are comparable in complexity and cost with the standard temperature detection method, its performances are much more susceptible to the quality of the Schottky interface. Inhomogeneities present on the contact surface, for either or both diodes, can significantly increase local current flow, leading to apparent effective area modifications, which then affect linearity and sensitivity consistency.

Finally, the dual diode, dual current levels (DDDC) approach finds a suitable compromise between the previous techniques by employing two identically-sized diodes, each biased at a different current. In this case, the differential voltage expression is identical to Equation (3). This method inherits the SDDC technique’s robustness to contact inhomogeneity influence through tuning of I_Fh_ and I_Fl_ values, while also allowing for the use of a simple and cost-effective readout circuit architecture. Sensor system performances are only noticeably affected by mismatches between either the dual diodes or their bias current sources.

Considering the advantages and drawbacks of the aforementioned temperature detection techniques, the DDDC method (with PTAT variation) was selected for implementation and comparison with the standard approach (single diode, biased at constant current, CTAT dependence), in the context of temperature sensing in a very wide range.

### 2.2. Sample Preparation

**Figure 1:**
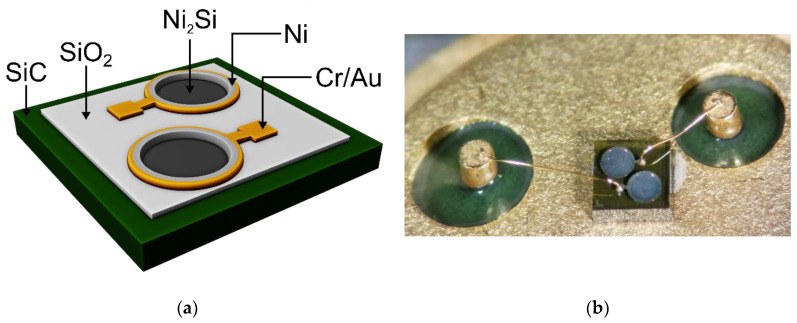
Dual-diode sensing element: (**a**) schematic illustration and (**b**) encapsulated structure.

The sensor structures consist of two SiC Schottky devices placed in close proximity (dual-diodes), with diagonal reverse symmetry, as is evinced in the Figure 1.

The fabrication process started from an n-type 4H-SiC substrate with 8 µm epitaxial layer, having~10^16^ cm^−3^ doping concentration. After a standard RCA [47] chemical cleaning, an initial dense layer of SiO_2_ (500 nm) was grown by Low Pressure Chemical Vapor Deposition (LPCVD), followed by a thermal annealing in O_2_ atmosphere at a temperature of 950 °C, for 30 min. Another SiO_2_ layer was subsequently deposited, without annealing, resulting in a less compact film. Next, circular active windows, with 400 µm diameters, were etched (using NH_4_F/CH_3_-COOH (180 mL/200 mL) solution in the oxide layers, resulting in a ramp profile termination [8,10]. This oxide ramp ensures a smooth current density distribution. After the active areas defining, the ohmic contact on the backside was obtained by deposition of a thin film of Ni (100 nm), followed by a rapid post-metallization annealing at a temperature of 1050 °C for 3 min in Ar atmosphere. Another thin film deposition of Ni (100 nm), in the active windows, was performed. A rapid post-metallization annealing at a temperature of 750 °C for 3 min in Ar atmosphere was carried out in order to obtain the Schottky contacts. Contact pads and the final back contact were finally defined after a deposition of (Cr (20 nm)/Au (300 nm)) on both sides of wafers. The test structures were diced into dual-diode chips and encapsulated in compact TO-39 packages using wire-bonding technology, as shown in Figure 1b.

### 2.3. Readout Circuit Architecture

**Figure 2:**
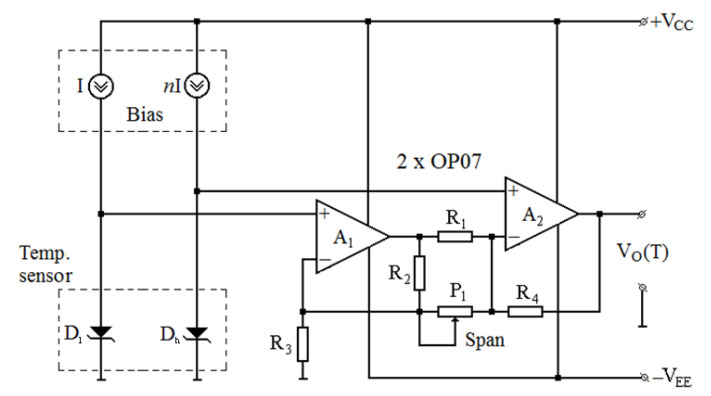
Schematic of the differential temperature measurement circuit.

The readout circuit is used to acquire and amplify the forward voltage difference given by the aforementioned dual-diode sensing element. It also biases the two identical diodes at different constant currents (the DDDC approach). Figure 2 represents the schematic of the proposed circuit. It includes a cost-effective, top-linearity instrumentation amplifier, comprising two low-noise, high-reliability OP07 [48] operational amplifiers, four resistors, and a potentiometer (P_1_-Figure 2) for output span tuning. The dual SiC Schottky diode structure is biased by an *I:nI Bias* block, based on the high-precision REF200 [49] current reference produced by Texas Instruments.

In order to greatly simplify the transfer function of the instrumentation amplifier, R_1_ = R_2_ and R_3_ = R_4_ is usually considered:

(5) $$V_{O}=\left(1+\frac{R_{3}}{R_{1}}+2\cdot \frac{R_{3}}{P_{1}}\right)\cdot \left(V_{Fh}-V_{Fl}\right)$$

For an optimum common-mode rejection, all resistors (R_1–4_) should be equal. As a result:

(6) $$V_{O}=2\cdot \left(1+\frac{R_{1}}{P_{1}}\right)\cdot \left(V_{Fh}-V_{Fl}\right)$$

The value of 10 kΩ was selected for these components in order to obtain a suitable compromise between power consumption, phase margin, and thermal noise. Because sensing diode voltages can reach low values at high operation temperatures, the circuit is powered by a ±15 V supply, which ensures good linearity over the entire output swing. This is the only noticeable difference between this topology, suitable for differential PTAT sensing, and the one described in [8], used for CTAT sensors. Otherwise, the two readout circuits are similar in complexity, cost, and gain tuning flexibility, offering high versatility over a wide array of temperature monitoring applications.

## 3. Results

### 3.1. X-ray Diffraction Analysis

**Figure 3:**
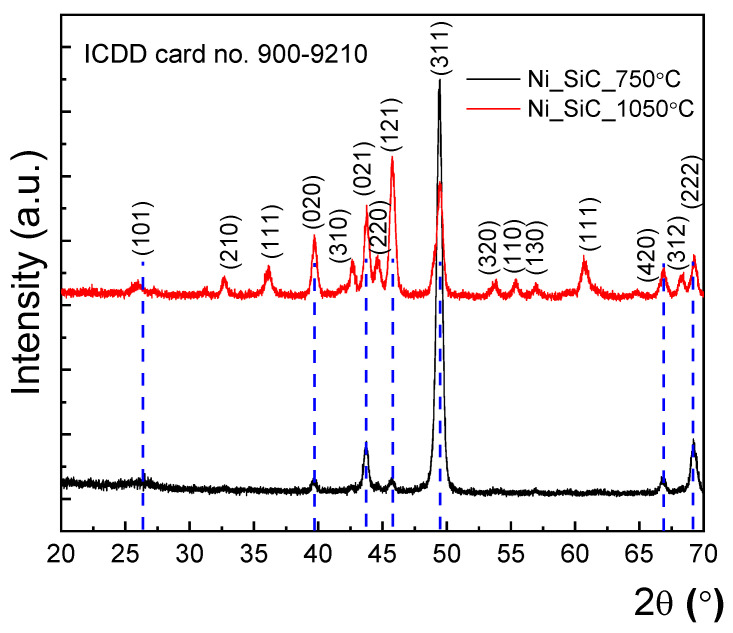
Grazing Incidence X-ray Diffraction (GI-XRD) patterns for the Schottky contact annealed at 750° (black line) and for the ohmic contact annealed at 1050 °C (red line), respectively. The dashed lines indicate the diffraction peaks position of the Schottky contact besides to the ohmic one.

In order to preliminarily assess the Schottky and ohmic contacts’ homogeneity, X-ray diffraction measurements were performed in grazing incidence geometry. The X-ray source was kept at 0.5°, while the detector scanned from 2θ = 20° up to 70°. A scan step of 0.01° at 4°/min was used for these investigations. Figure 3 presents the Grazing Incidence X-ray Diffraction (GI-XRD) patterns for both contacts.

As can be observed in Figure 3, both contacts present multiple diffraction peaks. These were assigned unambiguously as the Ni_2_Si phase, according to International Center for Diffraction Data (ICDD) database with card no. 900–9210 that belongs to the orthorhombic 62: Pbnm spatial group. Thus, the thermal treatment led to the formation of only the Ni_2_Si phase, without any additional phases. La Via et al. [50] report that a reaction between Ni and Si gives only the formation of the Ni_2_Si phase over a large annealing temperature range, between 600 °C and 950 °C. Later, the existence of a combination between Ni_31_Si_12_ and Ni_2_Si phases at 600 °C with an increase in Ni_2_Si percentage at 950 °C was also observed by XRD [51]. Kuchuk et al. [52] reported the formation of the Ni_2_Si phase as a result of a thermal treatment at 600 °C for 15 min. Increasing the annealing temperature further improved the occurrence rate of the NiSi_2_ phase. In our X-ray patterns, there are no traces of Ni or Si peaks, indicating that the thermally activated interaction between Ni and SiC had occurred. Moreover, taking into account the large area of the X-ray spot, namely 1 cm^2^, we conclude that there is no residual Ni on either contact. Regarding the crystal quality, the diffraction peak position of the Schottky contact, indicated with blue dashed line in Figure 3, was attributed to different Miller indices. There is a preferential orientation along the (311) direction. The increase in annealing temperature led to more pronounced polycrystalline features for the ohmic contact, which does not present a clear preferential orientation. The mean crystalline size was evaluated from a Williamson-Hall plot [53], with values of 20.1 nm (Schottky contact) and 19.6 nm (ohmic contact). Comparable crystalline domain sizes indicate that the thermal treatment did not induce the formation of additional structural defects. The XRD findings suggest that the thermal treatment led to Ni_2_Si phase formation on the entire Schottky and ohmic contact areas, for each of the dual-diodes. Additional dislocations were not generated.

### 3.2. Modeling and Sensing Performances

**Figure 4:**
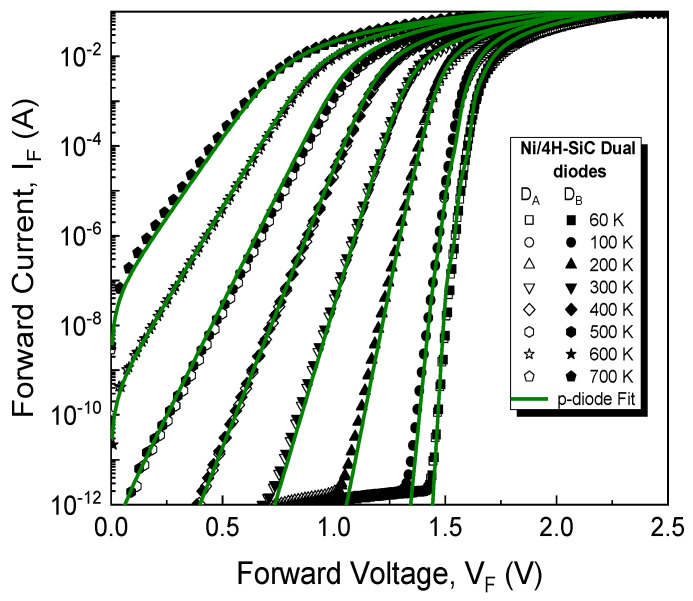
Forward I-V characteristic for the dual SiC Schottky diodes. Measurement data (symbols) and their p-diode model-fitted counterparts (lines).

The test samples were electrically characterized over a wide range of temperatures, starting from 60 K up to 700 K, with a step of 20 K. A Keithley 4200 Semiconductor Characterization System coupled with a Janis closed cycle refrigerator (CCS-450), capable of providing adequate means of cooling samples to temperatures below 77 K (liquid nitrogen), was used to perform measurements from 60 K to 500 K. For high-temperature measurements (300–700 K), the system described in [8] was used, comprising a Varian Chromatograph Oven and another Keithley 4200 SCS. Current-voltage (I-V) characteristics were acquired with the two systems on different days and experimental results were compared in the common 300–500 K interval to ensure reproducibility. The high-temperature stability of structures obtained with similar technological processes was demonstrated by thermal-cycling in [8]. Figure 4 depicts exemplary I-V-T characteristics for the dual diodes (D_A_ and D_B_). It can be seen that the devices have nearly identical forward electrical behavior over the entire 60–700 K domain. The exponential portion of the curves can be identified for each experimental characteristic, spanning at least five orders of magnitude, even at 700 K. For these reasons, it was considered that D_A_ and D_B_ both have the same Schottky contact parameters.

For the curves in Figure 4, the conventional barrier and ideality factor were extracted at each temperature, using the conventional method [54]. Φ_Bn,T_ was found to increase with temperature from 0.94 V to approx. 1.7 V, while *n* decreased from 1.85 to 1.01. These results indicate the presence of contact surface inhomogeneity. Thus, our recently introduced parallel-diode (*p-diode)* model was used in order to thoroughly characterize the sample, according to [40]:

(7) $$I_{F}=\overset{m}{\underset{i=1}{\sum }}I_{F,i}=A_{n}A_{S}T^{2}\overset{m}{\underset{i=1}{\sum }}\exp\left(\frac{-\Phi_{\mathrm{Bn},i}}{V_{\mathrm{th}}}-p_{eff,i}\right)\left[\exp\left(\frac{V_{F}-R_{S,i}I_{F,i}}{nV_{\mathrm{th}}}\right)-1\right]$$

The model assumes that an inhomogeneous contact comprises multiple regions which behave like ideal, parallel-connected diodes, each with associated barrier height (Φ_Bn,i_), non-uniformity parameter (*p_eff,i_*), near-unity ideality factor (*n* ≅ 1), and series resistance (R_S,i_). Out of the entire number of regions, only a few (counted by the model parameter *m*) contribute significantly to current conduction over the entire investigated temperature range. The *p_eff_* value is used to estimate the surface area for each of these essential regions [25,40]. Note that the total area occupied by the *m* regions can only be equal to or less than the nominal area (A_S_), with closer values indicating a better-quality diode.

In order to fully replicate the characteristics in Figure 4, *m* = 4 parallel-connected diodes (Dp1–Dp4) were necessary, with their model-parameters given in Table 1. Φ_Bn_ values are constant throughout the entire temperature range and tend towards the theoretical barrier height value for Ni_2_Si [25].

**Figure 5:**
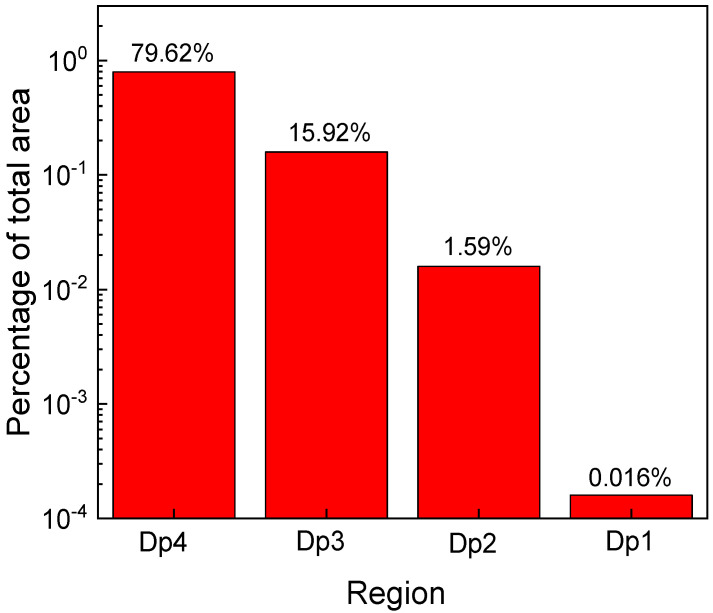
Parallel diode area percentages in respect to total Schottky contact area.

The values of *p_eff_* (Table 1) were estimated using Richardson plots over various temperature intervals and iteratively tuned, according to the technique proposed in [25,40]. Afterwards, they were used to determine occupied area percentages in respect to A_S_, for each parallel diode, as depicted in Figure 5. Note that nearly the entire Schottky contact surface is used for current conduction, especially at high bias and high temperatures.

**Figure 6:**
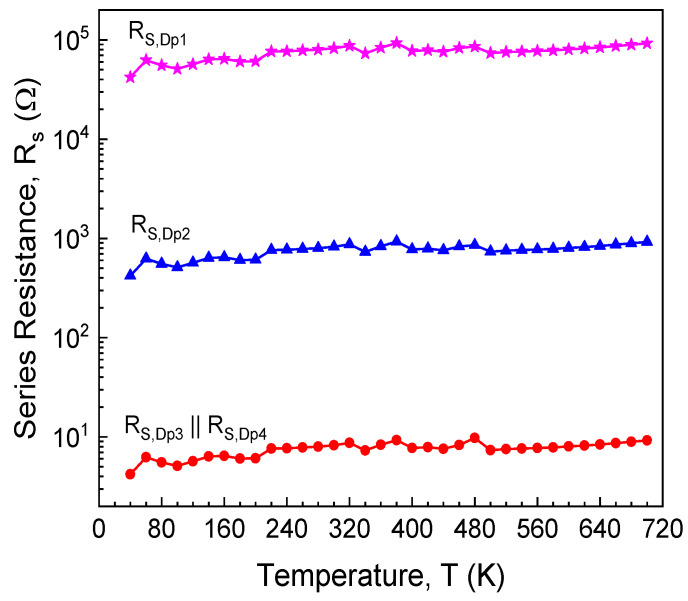
Series resistance temperature variation for the model parallel diodes (Dp1–Dp4).

Our model entails the evaluation of series resistance contributions [40]. Accordingly, the variations with temperature for the parallel diodes’ series resistances are illustrated in Figure 6. Because Dp3 and Dp4 have comparable current contributions at all temperatures, their individual series resistances could not be deconvoluted. Their combined ohmic behavior was determined, at each temperature, with Cheung’s method [54]. R_S_ values for Dp1 and Dp2 were adjusted to account for area differences, while keeping the same variation trend as R_S,Dp3_ || R_S,Dp4_.

The model-fitted forward curves, also depicted in Figure 4 (lines), are in good agreement with measurements for the entire temperature span. The fitting used Equation (7) with *m* = 4, the parameters from Table 1, and R_S_ data from Figure 6. Notably, the ideality factor, *n* = 1.01, was considered for each parallel diode, in the full 60–700 K domain. Dp3 and Dp4 significantly affect total current at all temperatures, especially in the high-bias region. Dp1 and Dp2 only influence conduction in the low-bias, low-temperature portion of the forward characteristics. Their impact becomes negligible past 200 K. For this investigated sample, our model [40] was able to completely reflect experimental forward behavior even at cryogenic temperatures (Figure 4). This is because, due to their area sizes which add up to almost the entire nominal surface (Figure 5), none of the parallel diodes suffers from the “pinch-off” effect [41]. This result attests to the high uniformity of the Schottky metal (Ni_2_Si, see Figure 3).

Analyzing the modeled electrical behavior of the dual-diodes (Figure 4, Figure 5 and Figure 6 and Table 1), a few conclusions can be drawn about their usability as temperature sensors:

- D_A_ and D_B_ may work over the entire domain of 60–700K, due to the localized effects of the parallel diodes. Forward bias-current values can be tuned to higher levels in order to restrict inhomogeneity influences (having only two parallel diodes dictate the majority current flow, rather than all of them).
- The standard CTAT sensing technique (using a single diode, either D_A_ or D_B_, biased at constant current) will suffer from poor linearity. This is because both Dp3 and Dp4 significantly affect conduction in the high-bias domain, but with different contributions depending on temperature.
- Using PTAT sensing techniques based on voltage difference can greatly improve linearity. As Dp3 and Dp4 have comparable barrier heights and effective areas, their combined apparent barrier height will have a slow temperature variation, which will be mitigated by forward voltage differentiation.

**Figure 7:**
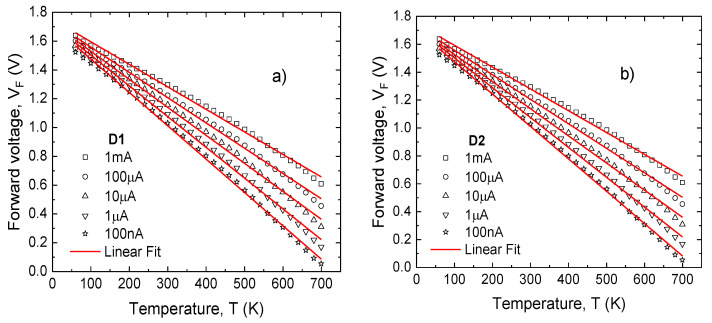
Forward voltage as a function of temperature for (**a**) D_A_ and (**b**) D_B_ at several bias currents.

Following these conclusions, an assessment of D_A_ and D_B_ temperature sensing performances was carried out. Their forward voltage variations with temperature, at different constant current levels, are presented in Figure 7, for each diode.

**Figure 8:**
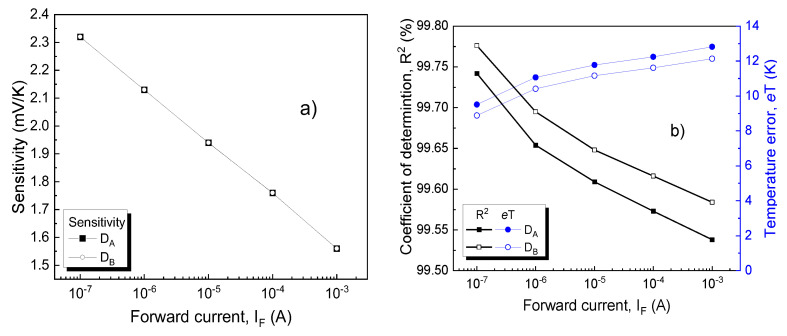
(**a**) Sensitivity, (**b**) coefficients of determination and average temperature error for both SiC Schottky diodes at different bias currents. For sensitivity, the absolute value was represented.

As expected, a CTAT dependence is obtained. For each individual device, a linear regression process on the characteristics in Figure 7 was performed. The slope of the fitted curves yielded the sensitivity. In order to assess linearity, the adjusted coefficient of determination (R^2^) was also determined. This parameter quantitatively evaluates how well a proposed model (in this case, a linear dependence) predicts experimental measurements. Additionally, for each current level, the fitting root mean squared error was divided by associated sensitivity in order to determine the temperature error (*e*T) [55]. As expected, *e*T varies complementarily to R^2^ [55]. Results are presented in Figure 8.

The sensitivities are nearly identical for D_A_ and D_B_ at all current levels, with a peak of 2.32 mV/K at 100 nA. Conversely, there are noticeable differences in linearity, favoring D_B_. Even so, the highest value for R^2^ is under 99.80%, below other reported results for such CTAT SiC-Schottky diode sensing elements [24,56]. These results confirm that extending the operation range to include both cryogenic and high-temperature levels, naturally impacts linearity (because of innate V_F_ variation, and contact inhomogeneity, as stated in Section 2.1).

**Figure 9:**
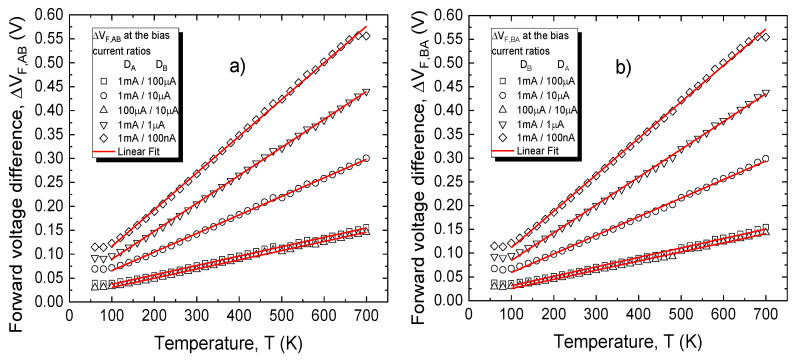
(**a**) ∆V_F,AB_ and (**b**) ∆V_F,BA_ with temperature, for different bias current ratios.

Temperature-sensing performances were also evaluated for differential setups corresponding to the DDDC approach. Thus, Figure 9 presents the temperature variation of both voltage differentiation possibilities (D_A_≡D_h_, when ∆V_F,AB_ = V_F,A_–V_F,B_ and D_B_≡D_h_, when ∆V_F,BA_ = V_F,B_–V_F,A_) at several bias current ratios. A PTAT variation is observed. In this case, temperature-sensitive electrical behavior could only be achieved in the 100–700 K interval.

**Figure 10:**
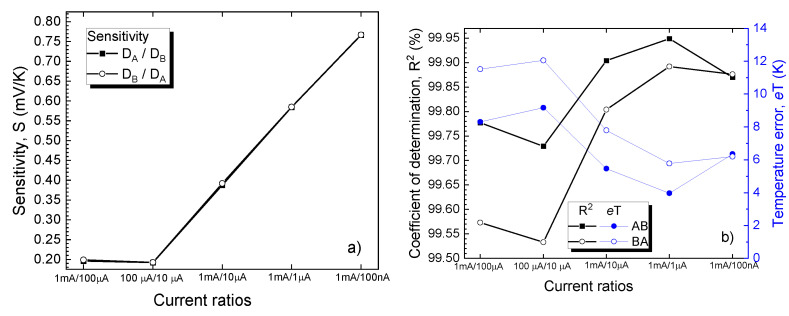
Sensitivity, coefficient of determination, and temperature error vs. diode current ratios for both differential configurations.

A linear regression process was also performed on the characteristics in Figure 9 in order to determine the sensitivity, linearity, and temperature error for the two DDDC configurations, with results plotted in Figure 10, for each current ratio.

Sensitivity values increase with current ratio and are nearly identical between the D_A_≡D_h_ and D_B_≡D_h_ cases. The maximum obtained was approx. 0.77 mV/K for the 1 mA/100 nA bias current ratio. On the other hand, linearity is affected by topology, with a peak R^2^ ≅ 99.95 % achieved for the D_A_≡D_h_ setup, at a bias ratio of 1 mA/1 µA. This result, together with a temperature error roughly three times lower, represent significant improvements over the CTAT variant (Figure 8b).

**Figure 11:**
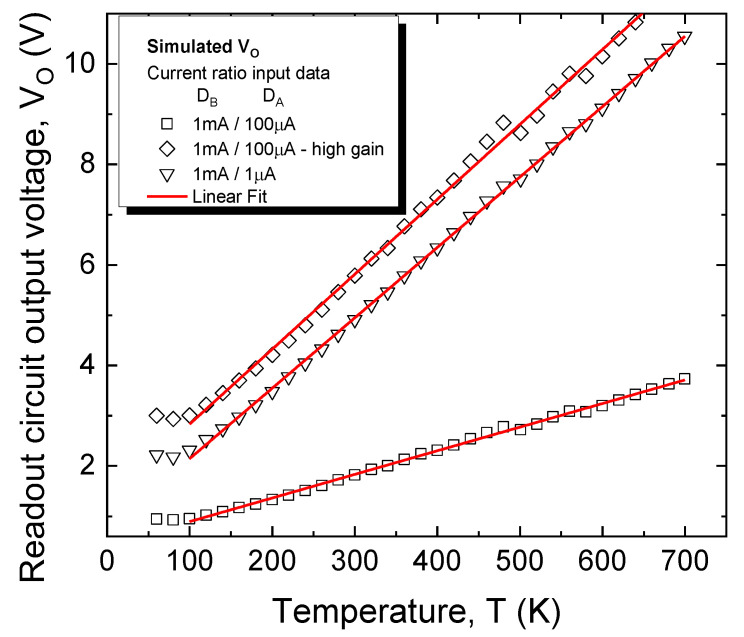
Simulated output voltage temperature dependence for the sensor system (dual-diode sensing element and readout circuit).

Overcoming the sensitivity loss of the DDDC approach can be achieved by using the circuit presented in Section 2.3. Investigations were carried out for the best-performing D_A_≡D_h_ configuration. In order to obtain the voltage-temperature dependence for the entire system, comprising both dual-diode sensing element and readout circuit, V_O_ as a function of input voltage difference was first simulated. P_1_ (Figure 2) was set to 910 Ω, resulting in a gain of approx. 24 (Equation (5)). The obtained transfer characteristics were composed with the data in Figure 9b for the 1 mA/100 µA and 1 mA/1 µA bias currents. The simulations were repeated for a higher gain of 76 (P_1_ set to 270 Ω) and again composed with the Figure 9b data. The resulting V_O_ (T) dependences are plotted in Figure 11, with sensing performances given in Table 2.

In all cases, adding the readout circuit significantly increases sensitivity, while keeping R^2^ virtually unchanged. Compared to the CTAT variant, using a gain of 24 is sufficient in order to match and even exceed sensitivity performances, without affecting linearity. Increasing the readout circuit’s gain further can compensate for S differences between bias current ratios; however, the mean squared error increases considerably (Table 2). Hence, only for the 1 mA/100 µA ratio, the gain could be increased to 76, which ensures maximum output swing for the readout circuit, while still maintaining linearity. The 1 mA/100 µA scenario was selected due to practical considerations. While it is obvious that the overall top results are attained for the 1 mA/1 µA case, obtaining accurate matching between such high-ratio current sources would notably increase the complexity and cost of the *I:nI Bias* block (Figure 2).

Sensing performances are summarized, alongside results obtained in recent papers, in Table 3. For a clear comparison, the sensing techniques are categorized separately. Sensitivity and linearity values are similar between considered contributions, with this work covering over double the working temperature interval.

## 4. Conclusions

This paper presented a dual-diode structure suitable for thermal sensing over very wide intervals, from cryogenic to high-temperature. It comprises two SiC-Schottky diodes with matched contact areas and symmetrical layout, placed in close proximity. These devices have nearly identical forward current-voltage characteristics, making them suitable for differential measurements. A highly uniform Schottky contact, covered with Ni_2_Si, was observed by XRD analysis on fabricated samples. Forward I-V-T measurements in the 60–700 K range evinced slight inhomogeneity. Thus, our p-diode technique was used to model the electrical behavior over the entire temperature domain, the largest reported so far. Two parallel diodes were sufficient in order to account for the majoritarian current at temperatures above 200 K. An additional two diodes were necessary for fitting lower temperature curves, at low-bias. The series resistance and effective surface of each parallel diode were taken into account in the model. Due to the large values obtained for these effective areas, the “pinch-off” effect was negligible, even at cryogenic temperatures.

The modeled dual-diodes proved suitable for CTAT and PTAT sensors over at least the 100–700 K range. For the CTAT variant, a high sensitivity was obtained (2.32 mV/K), with satisfactory linearity (R^2^ upwards of 99.80%) down to 60 K. Significantly better linearity was observed for the PTAT differential version, with R^2^ reaching 99.95%. In this case, the low sensitivity was overcome by using a high-performing, low-cost readout circuit. Simulations demonstrated sensitivities up to 14.91 mV/K, without affecting linearity.

## Figures and Tables

**Table 1:** Fitting parameters for sample S1.

Parallel Diode	Φ_Bn_ [V]	*p_eff_*	*n*
Dp1	1.56	8.75	1.01
Dp2	1.615	4.14	
Dp3	1.665	1.84	
Dp4	1.73	0.23	

**Table 2:** Sensor system performances.

Bias Current Setup		Gain	S [mV/K]	R^2^ [%]	Mean Squared Error [%]
$D_{A}\equiv D_{h}$	$D_{B}\equiv D_{l}$				
1 mA	100 µA	24	4.7	99.79	0.15
1 mA	100 µA	76	14.91	99.78	1.54
1 mA	1 µA	24	14.01	99.95	0.3

**Table 3:** Sensor system performance comparison.

	This Work	[39]	[46]	[55]
Sensing topology	Single SBD (CTAT)/Differential (PTAT)	Differential (PTAT)	Differential SBD/JBS (PTAT)	Single SBD (CTAT)
Temperature range	60–700 K (CTAT) 100–700 K (PTAT)	147–400 K	298–573 K	233–473 K
Sensitivity	2.32 mV/K (Single) 0.77 mV/K (Differential) 14.91 mV/K (Differential + Readout)	0.307 mV/K	4.32 mV/K (JBS) 2.85 mV/K (SBD)	3.425 mV/K
R^2^	99.8% (Single) 99.95% (Differential)	99.93%	99.96%	99.96%

## Data Availability

Not applicable.
